# Activation of atom-precise clusters for catalysis

**DOI:** 10.1039/c9na00549h

**Published:** 2019-11-07

**Authors:** V. Sudheeshkumar, Kazeem O. Sulaiman, Robert W. J. Scott

**Affiliations:** Department of Chemistry, University of Saskatchewan 110 Science Place Saskatoon Saskatchewan S7N 5C9 Canada robert.scott@usask.ca

## Abstract

The use of atom-precise, ligand-protected metal clusters has exceptional promise towards the fabrication of model supported-nanoparticle heterogeneous catalysts which have controlled sizes and compositions. One major challenge in the field involves the ease at which metallic clusters sinter upon removal of protected ligands, thus destroying the structural integrity of the model system. This review focuses on methods used to activate atom-precise thiolate-stabilized clusters for heterogeneous catalysis, and strategies that can be used to mitigate sintering. Thermal activation is the most commonly employed approach to activate atom-precise metal clusters, though a variety of chemical and photochemical activation strategies have also been reported. Material chemistry methods that can mitigate sintering are also explored, which include overcoating of clusters with metal oxide supports fabricated by sol–gel chemistry or atomic layer deposition of thin oxide films or encapsulating clusters within porous supports. In addition to focusing on the preservation of the size and morphology of deprotected metal clusters, the fate of the removed ligands is also explored, because detached and/or oxidized ligands can also greatly influence the overall properties of the catalyst systems. We also show that modern characterization techniques such as X-ray absorption spectroscopy and high-resolution electron microscopy have the capacity to enable careful monitoring of particle sintering upon activation of metal clusters.

## Abstract 2

This review details methods used to activate Au and Ag clusters for catalysis and routes that have been effective in mitigating sintering.

## Graphical abstract

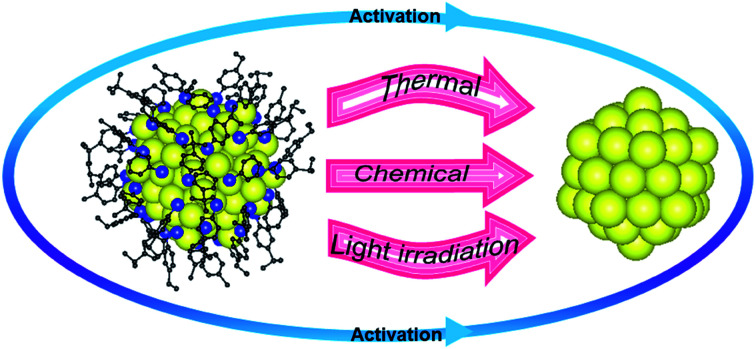

## 1. Introduction

Atom-precise, ligand-protected metal clusters, especially those comprising Au and Ag atoms, are receiving significant research attention owing to their excellent physicochemical properties which in turn enable their wide application in catalysis,^1–7^ biosensing,^8,9^ drug delivery,^10,11^ and biological imaging.^12^ While the term monolayer-protected clusters (MPCs) was used ubiquitously in the past for all thiolate-stabilized systems regardless of particle size, in this review we define atom-precise clusters as systems that typically have discrete electronic structures that vary with size, have well defined atom counts, and contain less than several hundred metal atoms and are below 2 nm in size. Such clusters have electronic properties that diverge significantly from those of larger nanoparticle systems which exhibit plasmon bands as a consequence of their metallic nature.

Catalysis by metal clusters has attracted tremendous research interest owing to advances in both the synthesis and characterization of atom-precise clusters. Synthesis of metal clusters is typically achieved by using ligands as stabilizing agents. Thiolate-stabilized metal clusters typically have a core–shell morphology with thiolate–Au–thiolate staple motifs protecting the core.^13^ Organic ligands such as thiols, acetylene, carbenes, phosphines, and selenolates not only provide stability to the metal clusters but also modulate the electronic states of the clusters.^14^ However, these stabilizers can inhibit the accessibility of active sites on the metal surface to the reacting substrate, which reduces catalytic activity.^15^ Past research work revealed that partial or complete removal of protecting ligands from the cluster surface improves the catalytic activity of metal clusters as catalysts. To achieve the activation of metal clusters, several approaches such as thermal calcination, chemical treatments with oxidizing or reducing agents, and light irradiation have been employed, albeit with the consequence of slight or significant particle size growth in some cases.

While there are many other reviews in the literature on the preparation and applications of atom-precise metal clusters as active catalysts for various chemical reactions, and others specifically on the roles of protecting ligands in the synthesis, properties, and catalytic activities of atom-precise Au clusters,^16–21^ this current work distinctly presents a review on recent advances in effective activation methods and approaches to significantly control sintering upon activation of atom-precise, ligand-protected Au- and Ag-based metal clusters. This review begins with a brief introductory section which is followed by the discussion of the synthesis, structure, and features of different atom-precise, ligand-protected metal clusters with emphasis on thiolate protected Au- and Ag-based clusters. Section 3 of this review focuses on thermal, chemical, and photochemical activation strategies to activate ligand protected clusters and the resulting structural integrity of the deprotected metal clusters. Finally, Section 4 discusses recent materials chemistry methods of creating overlayers on cluster surfaces or encapsulating clusters in porous supports to mitigate sintering upon activation of metal clusters. Thereafter, a summary of crucial lessons learned from the reviewed articles is shown, with some suggested future outlooks.

## 2. Ligand protected Au and Ag-based clusters

Noble metals with sizes on the nanoscale, also commonly referred to as nanoclusters, or simply as clusters (which is how they will be referred to in this review), generally show excellent catalytic activity due to their enhanced surface-to-volume ratio which leads to more active sites, as well as having modified surface geometries and tremendously different electronic properties as compared to bulk materials.^22^ Acknowledging the vast classes of metal clusters in the literature, this review focuses on atom-precise Au clusters, and to a lesser extent Ag clusters, as much more work has been done on these systems. Naked metal clusters are typically unstable in solution, and their syntheses and characterization become feasible when protected with small ligands such as thiolates, carbenes, phosphenes, and selenolates. The type of ligand, among other factors, influences reaction conditions for successful synthesis of ligand protected metal clusters. Although clusters protected with other ligands are included, emphasis is on different forms of thiolate-protected metal clusters. Thiolate protected clusters are widely studied due to strong sulfur–metal interactions that enable good stability in solution, facile synthesis, and controlled cluster compositions as well as functionalization of stable clusters.^23^

**Fig. 1:**
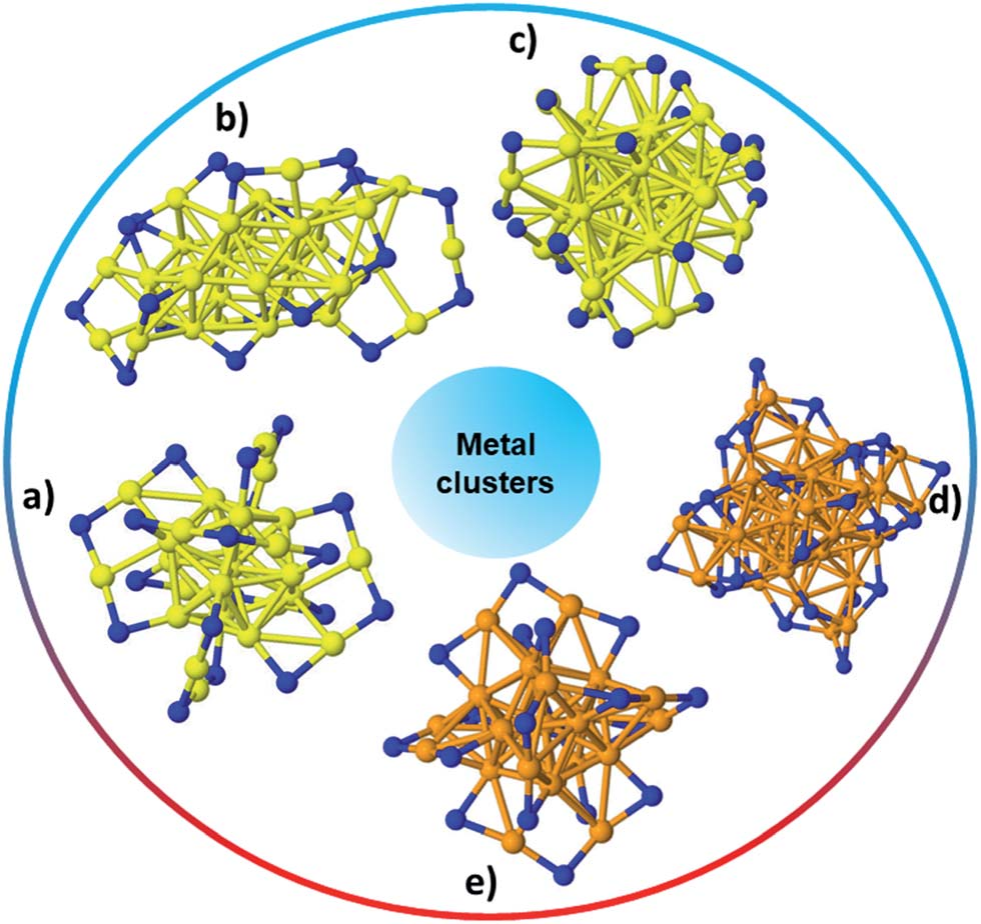
The structure of (a) Au_25_(SR)_18_,^35^ (b) Au_30_S(SR)_18_,^36^ (c) Au_38_(SR)_24_,^37^ (d) Ag_44_(SR)_30_,^40^ (e) Ag_25_(SR)_18_;^38^ Au: yellow, S: blue, and Ag: orange. Only the S atoms of thiolates are shown while the rest of the ligand has been omitted for clarity.

Metal clusters are an important class of materials due to their unique properties that differ from both their bulk and atomic counterparts. Among metal clusters, Au MPCs have been extensively studied. Brust and co-workers first reported a biphasic method in 1994 for synthesizing thiolate-based monolayer-protected clusters.^24^ In a typical Brust–Schiffrin synthesis of MPCs, the metal precursor is dissolved in an aqueous solution and transformed into the organic phase using phase transfer agents such as tetraoctylammonium bromide. In the second step, Au(iii) salts in toluene are converted into Au(i) species by reacting with thiol stabilizers. Finally, the Au(i) species are reduced by adding an excess of NaBH_4_. These Au MPCs were found to be relatively polydisperse in nature, and the size of the clusters could be tuned, to some extent, by changing the Au : thiol ratio and the type of thiol used. In recent years, tremendous research has focused on the ability to synthesize monodisperse, atom-precise metal clusters by optimizing the synthesis conditions such as the solvent, metal to thiol ratio, temperature, reducing agent, and purification and separation strategies.^17–19,25–28^ Atom-precise clusters are highly monodisperse, stable, structurally well-defined, and generally denoted as M_*x*_L_*y*_, where *x* is the number of metal atoms, and *y* is the number of protecting ligands (L) in the cluster composition. Many reports on the synthesis, characterization and applications of atom-precise thiolate ligand protected Au clusters such as Au_144_(SR)_60_, Au_102_(SR)_44_, Au_38_(SR)_24_, Au_25_(SR)_18_, *etc*., can be found in the literature.^29–32^ In recent years, many structures have been solved by single crystal X-ray crystallography, and clusters often have core–shell morphologies, wherein the Au core has certain geometrical structures that give unique physicochemical properties to the whole clusters while Au–thiolate staples cap the core structure.^33^ In 2007, the first crystal structure of Au clusters was published by Kornberg and co-workers, which was comprised of 102 Au atoms and 44 *p*-mercaptobenzoic acid ligands.^34^ In 2008, Murray *et al.* and Jin *et al.* independently reported the crystal structure of Au_25_(SR)_18_ clusters, which comprise an icosahedral Au_13_ core which is capped by six dimeric Au_2_(SR)_3_ staple motifs anchored on 12 out of 20 facets of the icosahedral core.^13,35^Fig. 1 shows the single-crystal structures of a number of commonly encountered thiolate-stabilized clusters in the literature; the formation of core–shell morphologies in which central cores are capped with metal–thiolate staples is ubiquitous throughout these structures.^35–39^

**Fig. 2:**
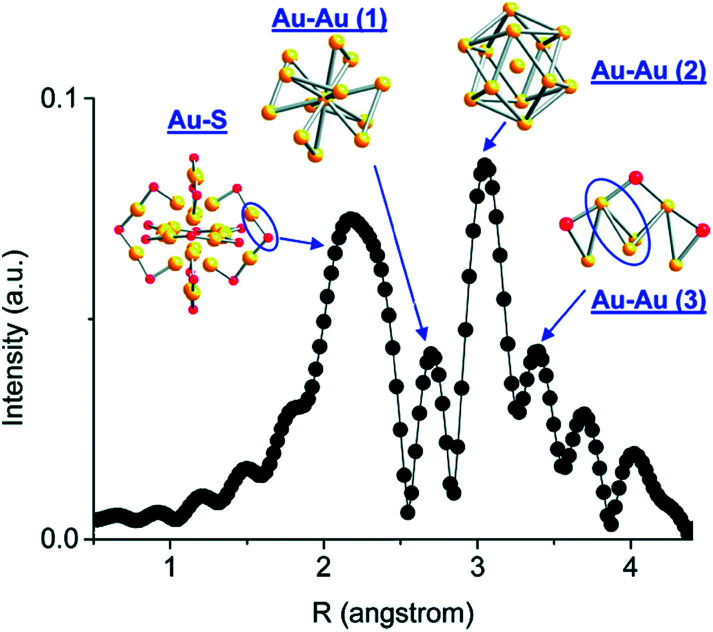
Simulated FT-EXAFS of Au_25_(SR)_18_ clusters. The simulation was done by averaging the EXAFS from all representative Au sites of Au_25_ (*k*: 3–14.5 Å^−1^, *k*^1^ weighted). The spectrum was phase-corrected using the Au–S peak. Reprinted with permission from ref. 43. Copyright 2011, American Chemical Society.

Atom-precise metal clusters have unique absorption behavior in the visible region of light, due to their discrete electronic structures.^41,42^ As a result, specific clusters show multiple features across the entire visible range in their optical absorption spectra that are defined by their core structures, and thus UV-Vis spectroscopy can be used as a facile technique, or fingerprint, to follow cluster speciation in solution. Mass spectrometry has been used in many cases to precisely follow the masses and charges on clusters. Ionization methods in mass spectrometry such as matrix-assisted laser desorption ionization (MALDI) and electron spray ionization (ESI) have allowed for the determination of the exact formulae of ligand-protected clusters, particularly in the absence of single-crystal X-ray crystallography data. Extended X-ray absorption fine structure (EXAFS) spectroscopy is another valuable tool that has been used to follow the structure of various supported and non-supported metal clusters. Based on the crystal structure, Zhang and coworkers demonstrated an atomic model of Au_25_(SR)_18_ clusters for EXAFS fitting.^43^ As shown in Fig. 2, the structure of Au_25_(SR)_18_ clusters is divided into several distinct bonding domains. The first prominent peak at approximately ∼2.3 Å is due to Au–S scattering. The first Au–Au contribution observed at ∼2.8 Å is due to the interatomic distance between the central Au atom of the icosahedral core and the 12 surface Au atoms. The second Au–Au interaction consists of bonds (∼2.95 Å) between the adjacent atoms on the surface of the icosahedral core. The last peak appears at ∼3.15 Å, which is due to the surface-staple Au–Au interactions.

Early work in the field suggested that Ag systems might behave significantly differently than analogous Au systems. Padmos and Zhang showed by XAS that as-synthesized small thiolate protected Ag nanoparticles seem to have Ag cores and Ag_2_S shells, while dialkylsulfide-stabilized Ag nanoparticles have pure Ag cores.^44^ Recently, Bakr and co-workers reported the successful synthesis and structural elucidation of Ag_25_(SR)_18_^−^ clusters that have similar (but not identical) atomic arrangements and ligand counts to Au_25_(SR)_18_^−^ clusters.^38^ Like Au_25_(SR)_18_^−^ clusters, Ag_25_(SR)_18_^−^ clusters have an Ag_13_ icosahedral core, but a more careful comparison of their crystal structures shows that there is difference in the arrangement of metal atoms around the core. In contrast to Au_25_(SR)_18_^−^, where all the twelve nonicosahedral Au atoms occupy the center of the triangular face centers of the Au_13_ icosahedral core, the Ag_25_(SR)_18_^−^ cluster has three of the nonicosahedral Ag atoms lying away from the triangular face centers, while the nine remaining nonicosahedral Ag atoms lay on the triangular face centers of the Ag_13_ icosahedral core. This kind of atomic arrangement enables Ag to be in the proximity of anchoring S of different v-shaped –S–Ag–S–Ag–S– motifs in the Ag_25_(SR)_18_^−^ clusters and thus facilitates weak intermotif interactions which are absent in Au_25_(SR)_18_^−^ clusters. Furthermore, the crystal structure of Ag_25_(SR)_18_^−^ has four larger voids that allow possible solvent coordination to give better stability of the nanoclusters, depending on the choice of the coordinating solvent. The preservation of all the distinct optical features in the UV-Vis absorption spectrum of the clusters in solution is often used to evaluate the stability of clusters in solution. We recently found that the values of the dielectric constant of the coordinating solvents correlates with the stability of Ag_25_L_18_^−^ clusters in solution and that lower temperatures (∼4 °C) enhance the stability of the clusters in solution.^45^

## 3. Activation strategies

Owing to the possible influences of the capping ligand on the activity and/or selectivity of ligand-protected metal clusters in catalytic reactions, it is desirable to have partial or complete ligand removal to enhance contact between the surface metal atoms and reactants, and thus allow higher catalytic activity. Common procedures entail immobilization of metal clusters onto support materials, followed by removal of ligands using a variety of activation strategies discussed in this section of this review. The method of immobilization and activation must be carefully chosen to avoid compromising the unique structure of the synthesized metal clusters. The challenge is to minimize cluster aggregation and sintering upon removal of protecting ligands from clusters loaded on solid supports. Available techniques to compare the sizes and distributions of clusters before and after ligand removal include high resolution transmission electron microscopy (HRTEM) and X-ray absorption spectroscopy (XAS). While methods of controlling sintering upon activation of clusters are discussed in the subsequent section, this section discusses recent advances in activation strategies/approaches for ligand-protected Au and Ag clusters.

### 3.1. Thermal approaches

One of the simplest approaches to activate atom-precise clusters involves the removal of ligands off metal surfaces by thermal calcination in air, which leads to the oxidation of ligands from the metal surface. However, in order to efficiently carry out such calcinations, it is important to be able to follow both the removal of the oxidized ligands and possible growth of the resulting activated clusters by sintering. In addition, in the case of some metal systems, it is possible that metal sulfide or oxide formation can occur during the calcination process.

Much early work towards understanding the thermal stability of thiolate-stabilized atom-precise clusters was carried out by Jin and coworkers, who examined the relative stability of Au–S binding modes in Au_25_(SR)_18_ (SR = glutathionate) clusters by NMR and optical spectroscopy.^46^ They found that ligands directly attached to the 13 atom Au core were more stable during thermal removal under nitrogen than the six thiolate ligands that were in the center of the staple motifs; the staple thiolates were removed at temperatures of 160 °C while the rest of the thiolates were stable until 180 °C. The thermal stability of Au_25_(SR)_18_ clusters was investigated by thermogravimetric analysis (TGA) under a N_2_ atmosphere at a ramp rate of 5 °C min^−1^. Significantly, they noted that these changes occurred even in the absence of any detectable mass loss by TGA analysis, which suggested that while the thiolates were removed from the Au surface, the ligands were still present in the final sample. This is important as it shows that TGA analyses themselves are not sufficient proof of structural integrity in such systems. Jin and coworkers subsequently performed TGA analyses of Au_25_(SCH_2_CH_2_Ph)_18_, Au_38_(SCH_2_CH_2_Ph)_24_ and Au_144_(SCH_2_CH_2_Ph)_60_ clusters.^47^ They showed that all the cluster samples begin to lose mass at a temperature of around 200 °C and all ligands were removed by *ca.* 250 °C. The calcined catalysts (200 °C for 2 h) showed a better catalytic activity for styrene epoxidation reactions than the uncalcined samples, which was likely due to the increased accessibility of the Au catalysts after partial ligand removal. However, moderate sintering was noted after thermal treatment at 200 °C for 2 h. The same group also studied the thermal decomposition of Au_144_(SR)_60_ clusters with various thiolate ligands.^48^ TGA analysis under a N_2_ atmosphere revealed that Au_144_ clusters protected by thiolate ligands with longer chains showed slightly higher stability, *i.e.*, Au_144_(SC_4_H_9_)_60_, Au_144_(SC_5_H_11_)_60_, and Au_144_(SC_6_H_13_)_60_ begin to show mass losses at 178 °C, 195 °C, and 205 °C, respectively.

**Fig. 3:**
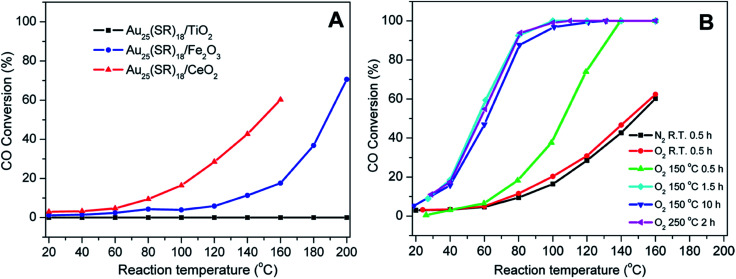
Activation of Au_25_(SR)_18_ clusters over different metal oxides for CO oxidation. (A) CO activity over different metal oxides as a function of temperature for unactivated clusters, and (B) CO activity on ceria supports under different activation conditions. Reprinted with permission from ref. 49. Copyright 2012, American Chemical Society.

Nie *et al.* examined the activation of phenylethanethiolate-stabilized Au_25_(SR)_18_ clusters on different oxide supports for CO oxidation, and found that optimal CO oxidation catalysts were generated using ceria supports and activation at 150 °C under oxygen, as seen in Fig. 3.^49^ They speculated that intact clusters were present as no mass loss was seen in the TGA at this temperature under oxygen. As the calcination time increased from 0.5 h to 1.5 h a drastic change in catalytic activity was observed as the CO conversion at 80 °C increased from 18.2% to 92.4%. However, no noticeable change in catalytic activity was seen as the pretreatment time was further increased from 1.5 h to 10 h, which indicated that thermal treatment for 1.5 h was sufficient for activation. Later, they observed that mild heating in the presence of an oxidative gas (O_2_) and reductive gases (CO or H_2_) mixture at 80 °C was more effective for the activation of Au_144_(SR)_60_/CeO_2_ catalysts.^50^ The catalytic activity studies for CO oxidation over the pretreated catalysts under different atmospheres (O_2_, O_2_/CO or O_2_/H_2_) revealed that an oxidative–reductive gas mixture plays an important role in boosting the CO conversion. The improvement in catalytic activity was explained by the formation of more oxygen vacancies on the ceria support after reductive gas treatment. Tsukuda and coworkers examined the activation of Au_25_(SR)_18_ clusters on hydroxyapatite supports, and showed that clusters activated at 300 °C could be used for the selective oxidation of styrene to styrene oxide.^51^ At this temperature, all thiolate ligands were removed from the sample as evidenced by the mass loss in the system. However, a slight increase in cluster size (1.4 nm) was noted in the activated catalyst. The Au_25_(SR)_18_ clusters on hydroxyapatite were found to be an effective catalyst for styrene epoxidation reactions.^51^ The activated clusters showed 100% conversion and 92% selectivity towards styrene epoxide using tertbutyl hydroperoxide (TBHP) as an oxidant in toluene at 80 °C. In other work, the same group synthesized hydroxyapatite-supported Au_*n*_ clusters (*n* = 10, 18, 25, and 39) and investigated the selective oxidation of cyclohexane to cyclohexanol and cyclohexanone.^52^ The glutathione protected Au_*n*_ clusters were deposited onto the support and then calcined at 300 °C for 2 h *in vacuo*. XPS and elemental analysis revealed the complete removal of glutathione ligands from the catalysts. During the calcination process, there was no significant change in cluster size as evidenced by TEM. The optimal cluster size for catalysis was found to be in the 39 Au atom range.

**Fig. 4:**
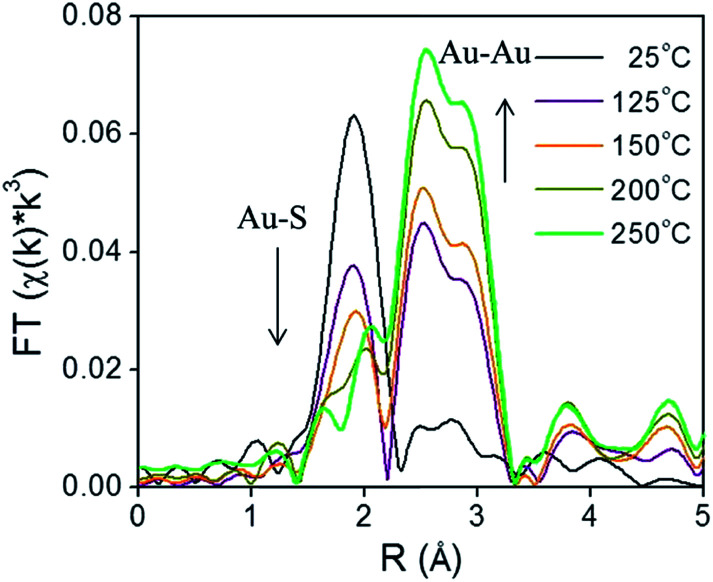
Au L_3_ edge EXAFS spectra in the R space of Au_25_(SR)_18_ clusters on carbon (with no phase shift correction). Reprinted with permission from ref. 54. Copyright 2013, American Chemical Society.

X-ray absorption spectroscopy (XAS) can be a valuable technique to follow cluster integrity upon calcination of supported-cluster materials. In an early study, Gaur *et al.* synthesized titania-supported Au_38_(SC_12_H_25_)_24_ clusters and activated the samples by calcination at 400 °C under a H_2_/He flow for 1 h.^53^ EXAFS analysis of catalysts before and after calcination gave clear evidence for the removal of thiols from the Au surface, as a peak due to the Au–S interatomic distance was observed around 2.3 Å in untreated and dried (100 °C for 1 h) catalysts, while the Au–S contribution was completely absent after thermal treatment. However, significant cluster sintering was seen in this system as the average Au particle size increased from 1.7 ± 0.2 nm to 3.9 ± 0.96 nm. Subsequently, our group reported a very careful study of the activation of phenylethanethiolate- and hexanethiolate-stabilized Au_25_(SR)_18_ clusters on carbon supports.^54^ Samples were calcined for 1.5 h in air at temperatures of 125 °C, 150 °C, 200 °C, and 250 °C and analyzed by EXAFS Au-L_3_ edge analysis. Fig. 4 shows the Au L_3_ edge EXAFS results for the phenylethanethiolate system. The results showed that the thiolate ligands start to be removed from the Au surface at 125 °C and were nearly completely removed from the Au surface at 250 °C. Importantly, no mass loss was seen in the TGA data until 150 °C. During the activation process, peaks due to Au–S species just below 2 Å slowly disappear, which indicates the removal of thiolate ligands. This disappearance of the Au–S peaks is accompanied by a growth in the first shell Au–Au peaks in the 2.5 to 3.0 Å region. EXAFS modelling shows that the coordination number (CN) of the Au–Au first shell contribution increased from 6.3(5) to 10.1(5) as the calcination temperature increased from 125 °C to 250 °C, which was strong evidence of Au cluster sintering. TEM images similarly showed that average particle sizes increased from 1.3 ± 0.1 nm to 1.9 ± 1.1 nm at these temperatures. The maximum activity for 4-nitrophenol reduction with NaBH_4_ was seen for clusters activated at 250 °C.

Wu *et al.* subsequently reported the activation of Au_25_(SR)_18_ clusters on ceria rods for CO oxidation.^55^ They noted that the thiolate ligands were a “double-edged sword” for CO oxidation as they blocked CO adsorption sites on Au while also being important to retain cluster integrity. Careful IR studies of CO adsorbed onto activated cluster surfaces showed that partially cationic (*δ*^+^) Au sites at the Au/ceria interface were likely the major catalytic sites for CO oxidation, and only appeared after calcination of the Au_25_(SR)_18_ clusters on ceria at temperatures of 150 °C and beyond. They also speculated that thiolate on–off dynamic states might be responsible for catalytic behavior in solution phase studies. Tsukuda and coworkers also showed that some ligand removal was essential for liquid phase aerobic oxidation of benzyl alcohol on Au_25_(SR)_18_ clusters supported on carbon nanosheet supports.^56^ They removed thiols by calcination under vacuum at temperatures between 400 and 500 °C, and found that ligands were increasingly removed at higher temperatures with little to no growth in cluster sizes. Interestingly, they found that Au clusters that were unactivated had no activity, while those that still had some residual thiolates were selective catalysts for the oxidation of benzyl alcohol to benzaldehyde, and the samples in which all thiolates were removed gave a much broader distribution of products (including benzoic acid and benzyl benzoate). The use of higher-temperature removal of thiols under vacuum needs to be investigated with other supports and thiolates to see if it is a general route to thiolate removal without significant cluster sintering.

A number of groups have also examined the role of the support in the resulting stability of Au clusters after activation. Yan and colleagues examined the activation of 6-mercaptohexanoic acid protected Au_25_(SR)_18_ clusters on various supports, and found that after calcination under nitrogen at 300 °C, no significant size growth of the clusters was seen on hydroxyapatite and Degussa P25 titania supports, while significant sintering of the clusters was seen on activated carbon, graphene oxide and silica supports.^57^ They postulated that the increased stability in the two systems was due to stronger interactions of the clusters with the supports in those cases; however it is not clear whether this result may be partially due to the use of 6-mercaptohexanoic acid ligands used in this system. Catalytic studies over calcined and uncalcined catalysts revealed that the removal of ligands from the Au surface was important as it enables the accessibility of the substrate. The pretreated Au clusters supported on hydroxyapatite and Degussa P25 titania catalysts showed more than 80% conversion for nitrobenzene hydrogenation reactions, while the uncalcined catalysts did not show any activity. García *et al.* examined the activation of Au_25_(SR)_18_ and Au_144_(SR)_60_ clusters over titania and silica supports, and found that Au_144_ clusters were more stable towards sintering than the smaller Au_25_ clusters, and both systems were more stable on silica supports.^58^

Another important variable in cluster sintering is the relative weight% loading of the clusters onto a support; one can possibly minimize sintering by ensuring optimum cluster loading onto supports prior to activation. For example, Xie *et al.* thermally activated Au_25_(SC_12_H_25_)_18_ clusters on multiwalled carbon nanotube supports at 300 °C and 400 °C for 2 h in a vacuum using Au loadings varying from 0.05–1.0 Au wt%. They reported that the optimum metal loading was 0.2 Au wt%, beyond which cluster aggregation became problematic.^59^ In another study, Lavenn *et al.* thermally activated Au_25_(SPh-*p*NH_2_)_17_ clusters supported on mesoporous silica SBA-15 at 400 °C while varying the metal loading (0.04 to 1.07 Au wt%), and reported similar average particle sizes: 1.9 ± 0.6 nm and 1.8 ± 0.5 nm for metal loadings of 0.04 and 1.07 Au wt%, respectively.^60^ The preservation of average particle size at higher loading could be partially due to the confinement of particles inside the mesopores.

**Fig. 5:**
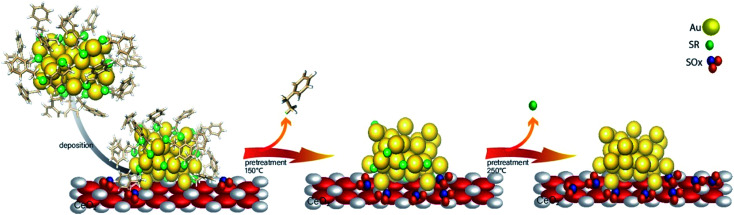
Thiolate ligand migration to the ceria support during the calcination process. Reprinted with permission from ref. 62.

While most research attention has been on the stability of metal clusters upon thiolate removal, very little work has focused on the fate of the removed ligands. This can be significant as oxidized ligands may still be present in the system after calcination, and thus can potentially modify the catalytic behaviour of the system. Zhang *et al.* examined the activation of Au_38_(SR)_24_ clusters on alumina and ceria supports in air and inert atmospheres.^61^ Cationic Au sites were observed on Au_38_/ceria samples calcined at 300 °C by Au L_3_ edge XAS, whereas these sites were absent when using analogous alumina supports. They also noted a two-step mass loss by TGA in air that was absent for samples heated under an inert atmosphere, which was possibly due to the different binding modes of the thiolates in the staples. In addition, upon using the resulting activated catalysts as cyclohexane oxidation catalysts, cyclohexanethiol was observed as one of the products, which showed that thiolate byproducts are still present on the support surface after activation. In a follow-up study, Zhang *et al.* observed ligand migration from Au_38_(SR)_24_ clusters to the ceria support after thermal treatments (Fig. 5).^62^ Sulfur K-edge XANES analysis clearly showed that thiolate migration not only leads to the formation of active sites on the Au surface but also leaves sulfur species such as disulfides, sulfites, and sulfates on the support. Recent work from the same group has noted the presence of SO_*x*_ species on the surface of the support during reactions.^63^ It was noted that the presence of these species can potentially limit the role of redox active supports in catalytic reactions. However, Alkmukhlifi *et al.* showed that while low levels of sulfates are present on inorganic support surfaces after the oxidation of supported thiolate-stabilized Au nanoparticles at 340 °C, the resulting catalysts are still active oxidation catalysts for hydrocarbon oxidation even with the sulfate present.^64^

While most of the above discussion has focused on thiolate-stabilized systems, there have also been a significant number of examples of thermally activated clusters using other ligand systems. The relative stability of Au_25_(SeC_8_H_17_)_18_*vs.* Au_25_(SC_8_H_17_)_18_ clusters during thermal calcination was explored by Kurashige *et al.*^65^ To probe the stability of these clusters against decomposition, TGA analysis was performed at a heating rate of 10 °C min^−1^ in a N_2_ atmosphere. The TGA curve starts to show mass losses at 136 °C for selenolate and 165 °C for thiolate ligands, which indicates that selenolate ligands begin to oxidize at a lower temperature than thiolate ligands. A number of groups have studied the activation of phosphine-stabilized clusters for catalysis. Tsukuda and coworkers investigated the catalytic performance of triphenylphosphine-protected Au_11_ clusters on mesoporous silica for benzyl alcohol oxidation.^66^ The phosphine ligands were removed by calcination at 200 °C for 2 h before the catalytic study. Wu *et al.* showed that Au_22_ clusters stabilized with six diphenylphoshine ligands (Au_22_(L)_6_) can oxidize CO without any ligand removal as evidenced by EXAFS and IR adsorption spectroscopy.^67^ They noted that uncoordinated Au sites in the intact clusters were able to absorb CO and activate oxygen. Wan and co-workers synthesized Au_38_ clusters with two different ligands Au_38_(L)_20_(Ph_3_P)_4_ (L = PhC≡C and 3-methylbenzenethiol) and studied the ligand effect on catalysis.^68^ In TGA analysis, the complete removal of thiolate ligands was observed at 250–300 °C, whereas PhC≡C ligands were removed completely at 400 °C. This result indicates that the phenylethynyl ligand is much more stable than the 3-methylbenzenethiol ligand during the thermal activation process. Anderson *et al.* published several papers examining the activation of various Au_*n*_(PPh_3_)_*y*_ (with *n* = 8, 9, 11, 101) clusters on titania nanoparticles by low-temperature calcination.^69,70^ They found that partial cluster sintering was seen after removal of phosphines at 200 °C heating in air. However, washing with toluene at 100 °C was shown to removal some of the phosphines with little to no aggregation of the clusters. Nakayama and coworkers similarly synthesized [Au_9_(PPh_3_)_8_](NO_3_)_3_ clusters and deposited them onto titania nanosheets.^71^ The activation of the clusters was achieved by calcination at 200 °C for 20 min under high vacuum. In XPS analysis, the P 2p_3/2_ peak disappeared after the thermal activation process which indicated the PPh_3_ ligand was removed from the system. However, tremendous cluster sintering, as evidenced by atomic force microscopy, was seen after phosphine removal.

Early work in the field suggested that Ag systems might behave significantly differently than analogous Au systems. Pradeep and coworkers found that glutathione-stabilized Ag_25_L_18_ clusters formed Ag_2_S materials, as evidenced by PXRD, heating at 80 °C in solution.^72^ However, later work by Tsukuda and coworkers employed XAS techniques to study the behavior of mesoporous carbon-supported [Ag_44_(SC_6_H_4_F)_30_]^−^ clusters upon thermal treatment.^73^ They observed sulfur-free Ag clusters upon calcination at 300 °C, which were used as catalysts for the catalytic dehydrogenation of ammonia borane. Our group recently employed XPS, XAS and other techniques to probe the thermal activation of 2,4-dimethylbenzenethiolate-protected Ag_25_ clusters on carbon supports.^45^ Our results showed that Ag-thiol bonds are selectively oxidized from the clusters upon mild heat treatments without formation of Ag_2_O or Ag_2_S, and that the activated Ag clusters on carbon supports showed particle size-dependent activity for styrene oxidation reactions. Specifically, XPS and EXAFS data showed that the resulting activated clusters are composed of Ag metal and that all thiols are removed from the Ag cluster surfaces; however XPS data showed that thiol oxidation products are still present in the sample, which is similar to the observation made by Zhang *et al.* which showed the migration of thiolate ligands from Au cluster surfaces to supports.^62^

### 3.2. Chemical approaches

#### 3.2.1. Oxidation (using O_3_, TBHP, KOH, *etc.*)

While oxidative calcination under air has been noted in the above section, a number of groups have examined alternative oxidants for cluster activation. Ozone exposure was found to be an effective method for the removal of stabilizing ligands from TiO_2_-supported Au_13_[PPh_3_]_4_[S(CH_2_)_11_CH_3_]_4_ clusters in order to active the clusters for CO oxidation.^74^ The ligand removal was achieved by flowing ozone (0.15% in oxygen) over the supported Au_13_ clusters at a rate of 1 ml min^−1^ for 1 h at room temperature. Both XPS and EXAFS analyses gave clear evidence for removal of ligands. This method provided considerable advantages over thermal treatment (400 °C for 2 h), which led to the particle size growth from 0.8 to 2.7 nm, whereas the post-ozone treated sample showed an average particle size of 1.2 nm. Hutchison and co-workers reported a slow oxidation process that precisely controls the exposure of the ligand shell to dilute ozone treatment, followed by the removal of the oxidized ligand by soaking in water.^75^ This strategy retains Au core sizes but suffers from incomplete removal of the ligand.

**Fig. 6:**
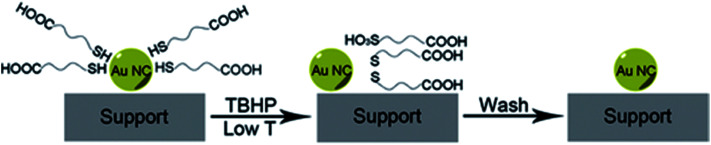
Soft oxidative removal of thiolates from Au clusters using peroxide oxidants. Reprinted with permission from ref. 79. Copyright 2015, John Wiley and Sons.

Peroxides have also been shown to be effective oxidizing agents for ligand removal. Kilmartin *et al.* observed that a strong oxidizing agent like tertbutyl hydroperoxide (TBHP) could be used to generate active Au catalysts from silica-supported Au_6_[(Ph_2_P-*o*-tolyl)_6_](NO_3_)_2_ clusters.^76^ While samples that were precalcined at 300 °C showed significant activity for the oxidation of benzyl alcohol with the peroxide, they noted that unactivated samples also began to be quite active after an induction period. Samples were heated up to 95 °C in benzyl alcohol in the presence of the peroxide, and gradual loss of the phosphine was observed over the first several hours of the reaction by Au L_3_ edge EXAFS. In early studies, several groups reported that unactivated thiolate-stabilized Au clusters were active for the oxidation of styrene with peroxides such as TBHP and oxygen gas.^47,77^ However, Dreier *et al.* noted that Au_25_(SR)_18_ clusters are not stable in the presence of peroxides under catalytic conditions, and control studies showed that mononuclear Au thiolate species that are removed from the cluster surface are likely the active catalyst.^78^ Poisoning experiments were done using phosphine additives as they noted that Au(i) phosphine systems were not typically active styrene oxidation catalysts. Similarly, Zhang *et al.* used TBHP to activate mercaptoalkanoic acid-stabilized Au_25_(SR)_18_ clusters that were supported on hydroxyapatite, as shown in Fig. 6.^79^ They noted that mercaptoalkanoic acid thiolates could be removed from the clusters as disulfide and sulfonate species at temperatures as low as 50 °C, while mercaptobenzoic acid ligands were not as easily removed. Significantly, the activation of the clusters using peroxide oxidants led to no significant increase in cluster sizes. Thus, the resulting activated clusters were much more active styrene and benzyl alcohol oxidation catalysts than clusters that were thermally calcined at 300 °C.

#### 3.2.2. Reduction (using LiBH_4_, NaBH_4_, *etc.*)

Another possible method to remove thiolates from metal cluster surfaces is to chemically reduce the thiolates from the surface, presumably as free thiols. This can be done, somewhat counterintuitively, using the same types of reducing agents as those used to make such clusters to begin with, *i.e.* using borohydride reducing agents. Typically, during syntheses, a large excess of thiol ligands is present in the reaction mixture, and any possible thiolate reduction and desorption are counterbalanced by the presence of large amounts of free thiols in solution. Both our group and others have shown that desorption of thiols occurs on purified Au–thiolate clusters in the presence of large excess NaBH_4_ concentrations. Dasog *et al.* showed that Au–thiolate bonds can be completely removed by concentrated strong reducing agents such as sodium borohydride, and the growth of monolayer-protected Au clusters (Au MPCs) can be controlled by changing the MPC : reducing agent ratio.^80^ Studies using alkanethiolate ligands with different chain lengths revealed that the immersion time for complete removal of thiols from Au surfaces becomes shorter when the chain length decreased. Ansar and coworkers demonstrated that removal of thiols from Au thiolate-stabilized nanoparticles could be achieved through thiolate displacement by NaBH_4_.^81^ They analyzed the kinetics of the thiolate removal from the Au surface by time-resolved UV-Vis measurements, and found that thiols could be completely removed using 25 mM NaBH_4_ for 10 min at room temperature. It was found that the rate of desorption can be accelerated by increasing the concentration of the reducing agent.

**Fig. 7:**
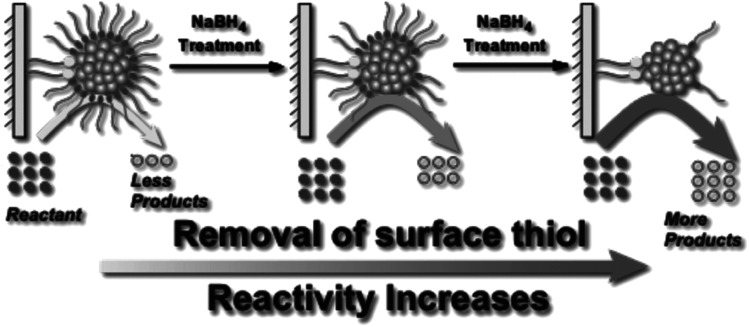
Illustration of enhanced catalytic activity in a selective oxidation reaction with supported thiolate-stabilized Au_25_(SR)_18_ cluster catalysts by mild chemical stripping of their surface ligands with NaBH_4_. Reprinted with permission from ref. 82. Copyright 2014 John Wiley and Sons.

Asefa and coworkers demonstrated that NaBH_4_ treatments of Au_25_(SCH_2_CH_2_Ph)_18_ and Au_144_(SCH_2_CH_2_Ph)_60_ clusters on mesoporous silica supports lead to an improvement in catalytic activity for styrene oxidation reactions, which was also attributed to the removal of thiolate ligands from the Au surface (Fig. 7).^82^ Our group studied the stability of Au_25_(SR)_18_ and larger Au_∼180_(SC_6_H_13_)_∼100_ clusters in high concentrations of NaBH_4_.^83^ Interestingly, Au_25_(SR)_18_ clusters in solution retained their structural integrity after NaBH_4_ treatments, whereas the larger cluster samples grew in size due to thiolate removal. However, the Au_25_(SR)_18_ clusters could be used as recyclable catalysts for the reduction of nitrophenol with NaBH_4_. In further work, we studied the advantages of chemical reduction treatments compared to thermal treatment for the activation of Au_25_(SR)_18_ clusters on alumina supports.^84^ Thiolate ligands were removed partially by treating alumina supported Au_25_(SC_8_H_9_)_18_ clusters with excess LiBH_4_ or LiAlH_4_ solutions. It was noted that some thiolate removal was seen by Au L_3_ edge EXAFS upon depositing the clusters on the alumina supports, which explains why the thiolates on supported clusters may be more easily removed than from clusters in solution. For samples calcined at 250 °C for 1.5 h in air, the supported clusters grew to an average size of ∼1.8 nm, while in contrast, cluster growth was inhibited when BH_4_^−^ reducing agents were used to remove ligands. Similarly, we have shown that bimetallic AuPd clusters can be activated on alumina supports by LiBH_4_ treatment with little to no growth of cluster size.^85^

### 3.3. Light-induced approaches

One area of intense research involving Au catalysts is the design of photocatalytically active materials by supporting Au clusters and/or nanoparticles on redox active metal oxide supports such as titania.^86–89^ A number of groups have noted that one can take advantage of the dye-like HOMO–LUMO transitions in Au_25_(SR)_18_ clusters to enhance visible light absorption for solar cell or photocatalytic applications.^90,91^ Yu *et al.* showed that unactivated phenylethanethiolate-stabilized Au_25_(SR)_18_ clusters on nanocrystalline titania can be used for the photocatalytic degradation of methyl orange.^91^ They noted that visible light could lead to the excitation of clusters followed by the transfer of excited electrons to the conduction band of titania, or alternatively, by the activation of oxygen by excited electrons in the LUMO of the clusters to form singlet oxygen. In the meantime, photogenerated holes in the HOMO can lead to the formation of hydroxyl radicals in aqueous solutions. Addition of a singlet oxygen quencher, l-histidine, led to a large decrease in activity. While the clusters were not activated before the reaction, there were no details provided on whether the thiolates present on the clusters remained intact during the photocatalytic process.

**Fig. 8:**
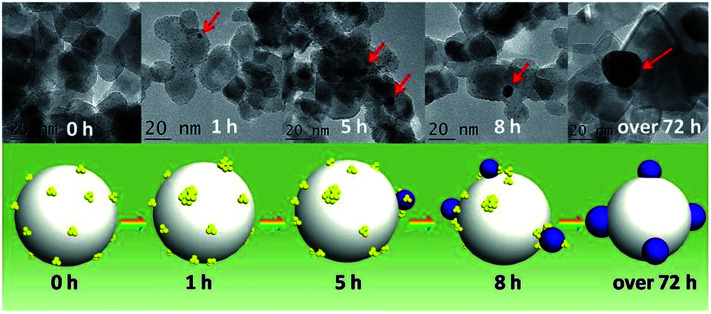
Illustration of the *in situ* transformation of Au clusters into Au nanoparticles on nanoporous titania nanotube arrays. Reprinted with permission from ref. 93.

Subsequent work by Liu and coworkers demonstrated that glutathione ligands were removed from *ca.* 1.5 nm Au clusters supported on TiO_2_ nanotubes by simulated solar light irradiation.^92^ A 300 W Xe arc lamp with an AM 1.5 cutoff filter and band-pass light filter (*λ* > 420 nm) was used as the light source. The complete transformation of Au_*x*_ clusters into Au nanoparticles was observed after 10 h of light illumination. The proposed mechanism for the transformation of Au clusters into Au nanoparticles under visible light irradiation involved photogenerated electrons in the clusters which enhances the reduction of Au(i) in the staple motifs to the metallic state. In addition, they noted that ligand removal may be facilitated by *in situ* formed active species such as hydroxyl radicals, superoxide radicals, and holes during the irradiation process. A similar report of light-induced cluster aggregation was reported by Liu and Xu for TiO_2_-supported Au_25_(SR)_18_ clusters during solar light irradiation using a 150 W Xe lamp.^93^ The thiolate ligand underwent an oxidation process which facilitates the transformation of Au_25_ clusters into larger Au nanoparticles, as shown in Fig. 8. The average Au nanoparticle sizes grew from *ca.* 1.3 nm to 3 nm, 7 nm, 10 nm, and 15 nm after irradiation for 1, 5, 8 and 72 h, respectively. Both hydroxyl and superoxide radicals were detected by electron spin resonance analysis under simulated solar light irradiation. Thus the reaction between photogenerated electrons and oxygen/water molecules leads to the formation of active intermediates such as hydroxyl/superoxide radicals which are responsible for the oxidative attack on the thiolate ligands. XPS studies of the sample before and after light irradiation gave clear evidence for the removal of thiolate ligands *via* the presence of sulfonate residues after illumination.

Light activation of Ag cluster systems has also been examined in the literature. Tatsuma and colleagues have shown that photoetching was seen for glutathione-stabilized Ag_32_(SR)_19_ clusters on titania when exposed to visible irradiation.^94^ AFM was used to follow cluster size changes as beam damage in TEM was noted to cause significant Ag cluster growth. A number of clusters were found to disappear under visible light irradiation. Meanwhile Abbas *et al.* have recently reported that Ag glutathione-stabilized clusters on titania coalesce into larger nanoparticles upon exposure to higher energy light.^95^ Initial particles were 2.2 nm in size while particles illuminated with a 387 nm laser at 30 mW cm^−2^ grew to an average size of 2.8 nm, albeit with a significant distribution of Ag sizes ranging from 1 to 8 nm. The authors speculated that simultaneous photoetching and coalescence likely occurred in this system.

## 4. Methods of controlling sintering upon activation of clusters

Sintering is the loss of the active surface area due to the agglomeration of nano-sized materials. As noted in the last section, sintering can be problematic in many scenarios that involve activating atom-precise clusters by using calcination and oxidation approaches, although some control of sintering was generated by selective removal of only some thiolate ligands from these systems or use of supports that promote strong support/cluster interactions. However, many important industrial reactions such as reforming of hydrocarbons, methane combustion reactions, and automobile exhaust control are carried out at higher temperatures (*i.e.* above 500 °C), and since many noble metal cluster and nanoparticle systems lack stability at such temperatures, industrial applications of such catalysts may be limited without further sintering control.

Encapsulation with metal oxide shells is a straightforward way of stabilizing metal nanoparticles towards sintering. This strategy involves the isolation of metal nanoparticles with a porous metal oxide shell such as silica, alumina, titania, or zirconia. For example, Somorjai and coworkers demonstrated that Pt nanoparticle sintering could be prevented by encapsulating Pt nanoparticles with silica shells which showed remarkable thermal stability even up to 750 °C.^96^ Silica shells with an average thickness of 17 nm were grown by sol–gel chemistry *via* the hydrolysis and condensation of tetraethylorthosilicate (TEOS) on tetradecyltrimethylammonium bromide protected Pt nanoparticles. However, the mass transfer issues associated with the metal oxide shell can be problematic in catalysis. Even though some metal oxide shells are porous, they may block a certain number of active sites on the surface of the catalyst. To overcome this mass transfer issue, Schüth and coworkers demonstrated another strategy to synthesize high-temperature stable Au nanoparticle catalysts with a yolk–shell structure.^97^ Au nanoparticles were encapsulated with a silica shell followed by a thin layer of zirconia using sol–gel chemistry. Finally, a yolk–shell structure around Au nanoparticles was created *via* selective etching of the inner silica layer.

**Fig. 9:**
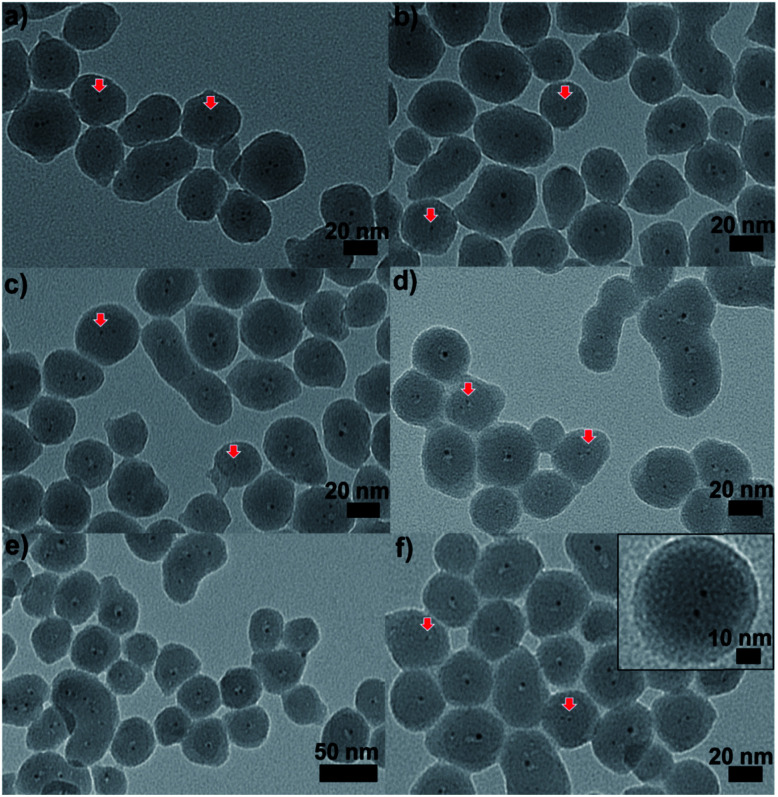
TEM images of Au_25_@SiO_2_ materials calcined at (a) 250 °C, (b) 350 °C, (c) 450 °C, (d) 550 °C, and (e) and (f) 650 °C (inset: enlarged image). Reproduced with permission of the Royal Society of Chemistry.^100^

There have only been a few examples of attempting to control sintering of atom-precise clusters by growing shells of metal oxides and other materials around the clusters.^98,99^ Samanta and co-workers showed that embedding arrays of multiple Au clusters (<2 nm) in a silica matrix could improve the thermal stability, in which clusters were encapsulated by silica.^99^ After calcination at 250 °C the size of the particle could be maintained below 3 nm. We reported that a protective silica shell grown by sol–gel chemistry with a thickness of 40 nm substantially enhanced the thermal stability of mercaptoundecanoic acid-protected Au_25_(SR)_18_ clusters, as shown in Fig. 9.^100^ The silica-encapsulated clusters showed tremendous sinter resistance upon calcination, and grew from *ca.* 1.1 nm ± 0.3 nm to 2.2 nm ± 1.0 nm and 3.2 ± 2.0 nm after calcination at 250 and 650 °C for 3 h, respectively. We believe that cluster growth occurred only due to aggregation of multiple clusters in some of the silica particles. Control samples of Au_25_(SR)_18_ clusters decorated on top of silica colloids showed tremendous sintering upon calcination with average particle sizes of 3.2 ± 1.7 nm and 15.5 ± 10.0 nm seen at similar calcination temperatures of 250 and 650 °C, respectively. However, mass transfer issues were identified in the final encapsulated clusters. Turnover numbers for styrene oxidation adjusted for the number of surface metal atoms suggested that encapsulated catalysts calcined at 650 °C were less hindered by mass transfer issues, potentially because all thiolate byproducts were removed at this temperature. Chen *et al.* reported an alternative method to improve the thermal stability of Au clusters by growing silica shells over Au_25_[SC_3_H_6_Si(OCH_3_)_3_]_18_ clusters.^101^ Au clusters were deposited on a silica core, and further layers were added by the hydrolysis of TEOS. The resulting materials showed improved sinter resistance, with average particle sizes of 2.0 ± 0.6 nm and 2.2 ± 0.5 nm after calcination at 400 and 600 °C, respectively. A fraction of >4 nm Au nanoparticles were seen at the higher calcination temperature as some Au nanoparticles were able to escape the silica shells, and as a result the resulting samples calcined at 600 °C showed slightly lower activity for 4-nitrophenol reduction than that seen for samples calcined at 400 °C.

Another method to improve sintering control is to isolate clusters within two dimensional mesoporous materials. In an early example, Dai and colleagues showed that Au_25_(SR)_18_ and Au_144_(SR)_60_ clusters could be stabilized towards sintering by incorporating them into mesoporous silica that was coated with CuO.^102^ Clusters on pure mesoporous silica showed tremendous sintering after calcination at 300 °C, whereas those that were deposited onto CuO intermediate layers had an average size of 1.67 ± 0.2 nm after calcination at the same temperature. Similar results were obtained for Co_3_O_4_ overlayers on silica. Control studies of clusters deposited on similar oxides on non-porous silica supports showed poorer sintering resistance. Lavenn *et al.* thermally activated Au_25_(SPh-*p*NH_2_)_17_ clusters supported on mesoporous silica SBA-15 at 400 °C and reported that the Au clusters grew to just under 2 nm after calcination.^60^ Thus, there is some evidence that ordered porous silica templates can partially mitigate sintering, but work better in the presence of a secondary stabilization mechanism. Several groups have examined the use of thiol tethers on the surface of porous silica materials to anchor Au clusters, followed by activation. For example, Das *et al.* showed that 3-mercaptopropyltrimethoxysilane (MPTS) could be anchored onto mesoporous SBA-15 silica, which allowed for efficient anchoring of Au_25_(SR)_18_ and Au_144_(SR)_60_ clusters. This was followed by activation of the clusters by partial chemical removal of thiolates with NaBH_4_.^82^ Similarly, Zheng *et al.* showed that porous silica spheres decorated with MPTS could capture Au clusters. The resulting materials showed moderate sintering resistance when samples were activated under a H_2_ atmosphere at 350 °C.^103^

Xu and coworkers reported a method to improve the photostability of glutathione protected Au clusters on silica spheres using branched polyethylenimine for surface modification.^104^ The structural integrity of Au clusters was preserved after 10 h light irradiation (420 nm), likely because the surface modification prevents the glutathione ligands from oxidizing. An additional coating with a titania shell further improved the photostability of these clusters. Sintering of glutathione protected clusters could be eliminated to some extent by encapsulation within a metal–organic framework, which has been reported by Xiong and coworkers.^105^ Xu and coworkers reported that hydroxyl groups on the surface of titania have a critical role in the stability of Au clusters during the light irradiation process.^106^ Hydroxyl radicals could be created by the interaction of surface hydroxyl groups and photogenerated holes, which leads to the decomposition of protecting ligands around the cluster, resulting in clusters sintering to form Au nanoparticles. It was observed that the replacement of the hydroxyl groups with fluoride ions enhanced the photostability of the clusters.

**Fig. 10:**
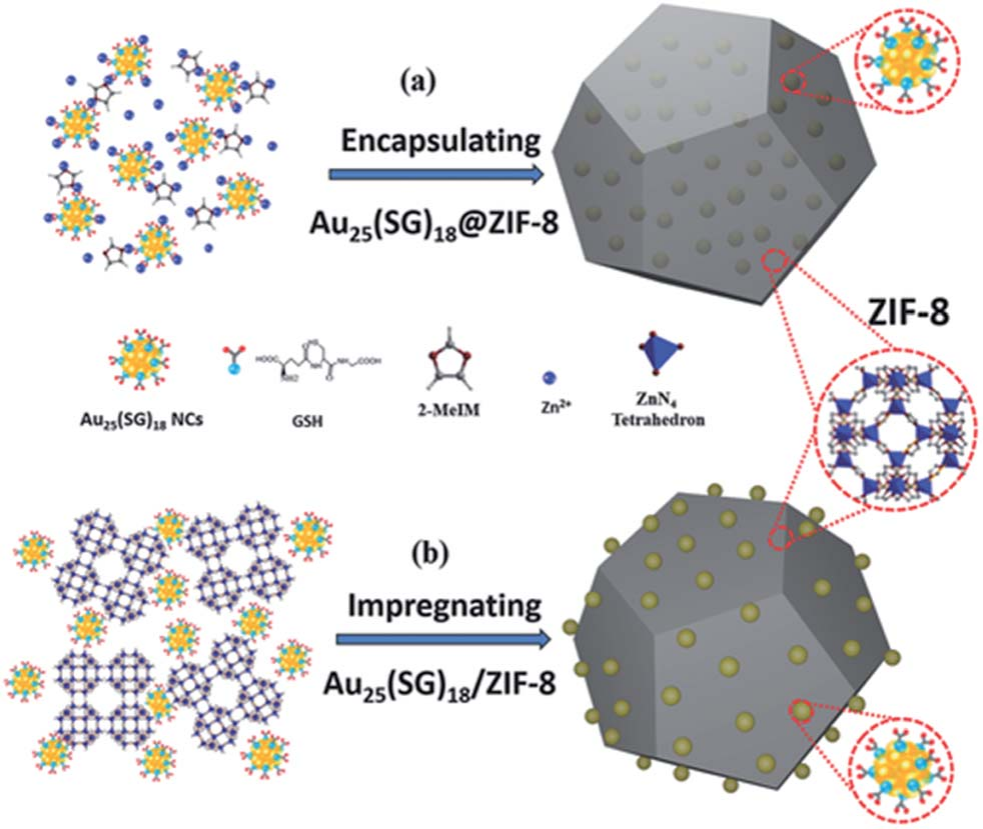
Illustration of Au_25_(SR)_18_ clusters (a) encapsulated in, and (b) impregnated onto, a ZIF-8 metal–organic framework. Reproduced with permission from ref. 110. Copyright 2018 John Wiley and Sons.

Physical confinement within metal–organic frameworks (MOFs) provides a novel strategy for improving the thermal stability of clusters. Zhu and coworkers detailed the synthesis of Au_11_(PPh_3_)Cl_2_ and Au_13_Ag_12_(PPh_3_)_10_Cl_8_ clusters inside ZIF-8 (Zn(2-methylimidazole)_2_) and MIL-101 (Cr_3_F(H_2_O)_2_O(1,4-benzenedicarboxylate)_3_) templates.^107^ The authors noted that not all of the clusters were encapsulated in the MOF; a fraction of the clusters were formed on the MOF surface. The systems could be activated for catalysis at 150 °C; however moderate sintering was observed after calcination of the MOF/cluster composites at temperatures of 200 °C and beyond. In further work, the same group demonstrated an electrostatic attraction strategy to incorporate [Au_12_Ag_32_(SR)_30_]^4−^, [Ag_44_(SR)_30_]^4−^, and [Ag_12_Cu_28_(SR)_30_]^4−^ nanoclusters within ZIF-8, ZIF-67, and manganese hexacyanoferrate hydrate frameworks by a cation exchange strategy.^108^ Similarly, Rosi and coworkers showed that cationic Au_133_(SR)_52_ clusters could be incorporated into the surface of MOF crystals by cation exchange.^109^ However, the thermal stability of these systems was not analyzed in either of these publications. Luo *et al.* reported an approach for improving the thermal stability of clusters by embedding glutathione-stabilized Au_25_(SR)_18_ clusters in a ZIF-8 metal–organic framework, as shown in Fig. 10.^110^ Two nanocomposites were synthesized by incorporating clusters either inside or outside of the framework. Au clusters were encapsulated into the framework *via* ‘coordination assisted self-assembly’ or alternatively decorated on the MOF surface by impregnation of clusters onto the surface of the framework. After calcination at 300 °C in a nitrogen atmosphere, both systems did not show notable aggregation and maintained the dispersity of Au clusters in or on the MOF. For the comparison of the properties of these two systems, the catalytic activity for the 4-nitrophenol reduction reaction was studied. Au_25_(SR)_18_ clusters within the MOF showed less activity than those on the surface of the MOF, which is likely due to mass-transfer issues of the substrate accessing clusters within the MOF.

**Fig. 11:**
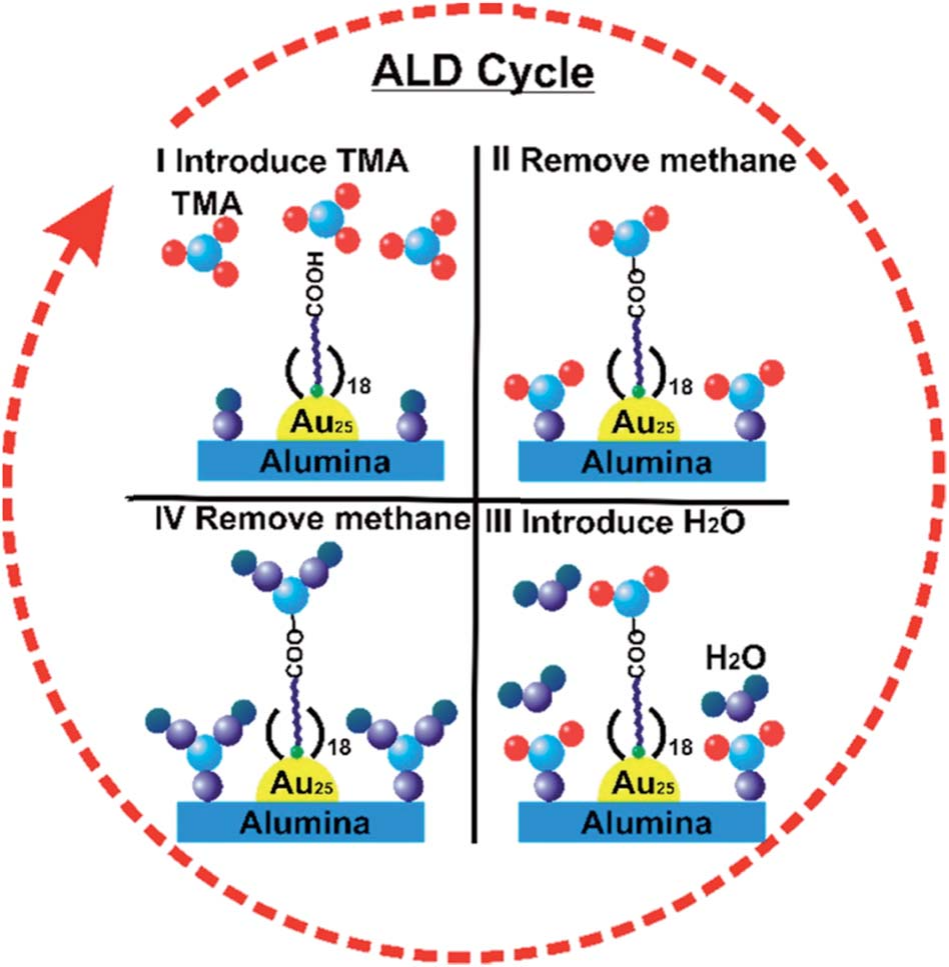
General scheme of alumina deposition over Au_25_(MUA)_18_/Al_2_O_3_ by atomic layer deposition. Reprinted with permission from ref. 111. Copyright 2018 American Chemical Society.

Atomic Layer Deposition (ALD) is a unique method for fabricating a thin layer of metal oxides over nanomaterials. We recently reported that the nature of the ligand on Au clusters has a large role in the effectiveness of ALD overlayer growth.^111^ As shown in Fig. 11, alumina overlayers were deposited on Au_25_(SR)_18_ clusters protected with two different ligands (mercaptoundecanoic acid and dodecanethiol), and dramatically improved thermal stability of clusters was seen for clusters which had surface carboxylic acid groups. This is likely due to the fact that the trimethylaluminum ALD precursors can anchor to surface carboxylate groups, leading to ALD overlayers on top of the clusters in that system, while for dodecanethiolate stabilized clusters, ALD growth can only occur around the clusters. To study the effect of the thickness of alumina layers on thermal stability, catalysts were synthesized by 5, 10, and 20 cycles of alumina deposition over Au_25_(MUA)_18_ (MUA = mercaptoundecanoic acid) clusters predeposited on alumina supports. Au_25_(MUA)_18_ clusters stabilized by 20 cycles of alumina overcoating were much more sinter-resistant than 5 and 10 cycle-coated clusters. The average particle size of Au_25_(MUA)_18_ clusters coated with 20 cycles of alumina overcoating calcined at 250 °C and 650 °C was found to be 1.8 ± 0.5 nm and 2.4 ± 0.9 nm respectively.^111^

## 5. Summary and outlook

The presence of protecting thiolate ligands on metal clusters enables the synthesis of well-defined structures and influences the physicochemical and catalytic properties of metal clusters. As shown in this review, the deprotection of metal clusters without growth in size is commonly desirable in establishing the size-dependent catalytic performance of metal clusters; however, having ligands intact on metal clusters can be beneficial in some cases. For instance, Zhao *et al.* showed that having intact ligands on Au_38_(SR)_22_ clusters enabled chemoselective hydrogenation of aldehyde groups of nitrobenzaldehyde derivatives, while deprotected Au clusters favored hydrogenation of nitro groups.^112^ While the activation of metal clusters is expected to enable enhanced contact between metal atoms and reactants, the activation conditions must be carefully selected to prevent particle agglomeration and sintering. In addition, thermal gravimetric analysis alone cannot be used to adequately prove the preservation of the structural integrity of metal clusters, as thiolate ligands can be removed from the metal surface but still present in the final sample. Thus, other complementary techniques like XAS and XPS should be employed in monitoring the removal of protecting ligands from the metal surface.

Thermal activation remains the simplest and most studied approach. The use of vacuum-assisted removal of thiols from Au clusters supported on carbon nanosheet supports resulted in little or no growth in cluster sizes but this method needs to be further investigated with other supports to establish whether it is a general route to thiolate removal without significant cluster sintering, as supports generally play a major role in stabilizing metal clusters after activation.^58^ While not an atom-specific cluster example, a general strategy towards organic ligand removal which minimizes sintering was shown by Cargnello and colleagues, who showed that a variety of metallic nanoparticles could be activated with no sintering by shock treating samples in air using exceptionally fast heating and cooling ramps (<1 minute).^113^ They postulated that this allowed for kinetic transformations to take place rather than thermodynamic transformations, and thus removes the thermodynamic driving force for nanoparticle aggregation. It would be interesting to see if this general methodology works for atom-precise clusters as well. Aside from oxidative calcination under air, the use of alternative oxidants is another common method for cluster activation. Mild ozone treatment provides considerable advantages over harsher thermal treatments (400 °C for 2 h) for mitigating cluster sintering. Meanwhile, peroxides have shown to be effective oxidizing agents for ligand removal without a significant increase in cluster sizes. Counterintuitively, thiolate ligands could be chemically reduced, presumably to free thiols, using the same types of reducing agents employed in the synthesis of protected metal clusters. In the case of alkanethiolate ligands, the immersion time for complete removal of thiol from the Au substrate correlated with the chain length of the ligand. It was speculated that thiolates on supported clusters might be more easily removed than from clusters in solution. Also, both visible and UV light have been employed for activation of thiolate-protected metal clusters, and the light-induced oxidation of thiolate ligands resulted in metal cluster aggregation to form metal nanoparticles.

Control of particle aggregation and sintering upon deprotection of metal clusters on solid supports can be achieved by creating physical barriers and/or providing strong metal support interactions. Encapsulation with metal oxide shells results in enhanced thermal stability of metal clusters but introduces mass transfer issues associated with metal oxide shells.^100,111,114–116^ Another effective strategy of controlling sintering entails incorporation of metal clusters into mesoporous silica coated with metal oxides, whereas ordered porous silica templates themselves do not completely mitigate sintering without secondary stabilizers. Similar physical confinement within metal–organic frameworks offers improved thermal stability. The ALD technique enables fabrication of a thin layer of metal oxides over metal clusters but the nature of the protecting ligand on metal clusters plays a major role in the effectiveness of ALD overlayer growth. Despite this progress in designing sinter-resistant clusters, complete stabilization of clusters against sintering is yet to be attained in most cases as minor sintering, due to the agglomeration of adjacent clusters, is still problematic. Thus, it is necessary to develop new methodologies that can ensure excellent stabilization to maintain the structural integrity of these clusters during the activation process. Moreover, many studies have only focused on thermal stability with little attention being paid to the mass transfer issue which is associated with a protective shell. The enhanced thermal stability of encapsulated metal clusters is often achieved at the cost of their catalytic activity, owing to the blocking of active sites and delayed mass transport. Zhan and coworkers demonstrated that particle sintering and surface blockage associated with organic moieties around nanoparticles can be eliminated by *in situ* carbonization of these ligands and the resulting carbon shell can serve as a physical barrier against sintering.^117^ By annealing at 500 °C under a nitrogen atmosphere, the protecting ligands around Au nanoparticles carbonized into a shell that offers partial encapsulation and this may be an efficient way of significantly slowing down the sintering of clusters while maintaining the exposure of active sites.

Besides the stability of deprotected metal clusters, research attention should also be focused on the fate of the removed ligands, as the oxidized ligands can potentially modify the catalytic behaviour of the system.^62^ Importantly, having the same ligand type on metal clusters of different sizes will enable studies that elucidate structure–property relationships. While some examples of such relationships have been developed for Au clusters, such a study is yet to be reported for Ag and other metals as different atom-precise metal clusters with the same ligand type are difficult to successfully prepare. Following cluster transformation *via in situ* studies may give valuable knowledge about how atomic rearrangements occur as clusters are activated – do metal atoms in the staples become part of the underlying core, or are they cleaved to form separate atomic species on the surface, which can then undergo separate nucleation and/or growth events? It would be useful to find routes to selectively remove staples from clusters without affecting the underlying core. For example Black *et al.* have recently investigated the use of high energy UV light to efficiently strip ligands from Au_25_(SR)_18_ and Au_36_(SR)_24_ clusters.^118^ Ultraviolet photodissociation mass spectrometry measurements showed that single high energy ultraviolet pulses (*λ* = 193 nm) could cause extensive stripping of ligands off the staple motifs without removing any of the underlying Au atoms, while five or more pulses resulted in the formation of Au_4_(SR)_4_ tetramers and Au atom loss from the clusters. While this essentially is a pure mass spectrometry report, the use of high energy ultraviolet light pulses to remove ligands would be a novel concept towards activating clusters on supports.

While this review has focused on Au and, to a much lesser extent, Ag clusters, there is a large amount of current research being done on atom-precise bimetallic clusters.^28,59^ Such systems are of particular research interest as such subtle modification of a cluster by a single dopant atom can result in significant synergistic enhancements in the catalytic efficiency, depending on the type of dopant atom. For instance, several studies have shown that a single atom doping of Au_25_ leads to both improved cluster stability and enhanced catalytic efficiency.^59,119–122^ Similar improvement in stability and catalytic performance has been observed for atom-precise Ag-based bimetallic clusters.^123–126^ While most of the existing studies have reported the catalytic activities of bimetallic clusters with protecting ligands intact, Xie *et al.* reported the enhanced catalytic activity of thermally activated Pd_1_Au_24_(SC_12_H_25_)_18_ clusters on multiwalled carbon nanotube supports.^59^ Their results showed that no activity was found for unactivated Pd_1_Au_24_(SC_12_H_25_)_1_ and Au_25_(SC_12_H_25_)_18_ systems, but activated catalysts were active for aerobic oxidation of benzyl alcohol. Single Pd atom doping significantly enhanced the catalytic performance of activated Au_25_ clusters. However, the study did not thoroughly establish the preservation of the structural integrity of the bimetallic clusters upon activation. Thus more studies are needed towards determining the preservation of the structural integrity of bimetallic clusters upon activation, as it is likely that sintering, metal oxidation, and phase separation will be issues, particularly for the activation of bimetallic systems at high temperatures.

Overall, there has been tremendous progress in the field towards the design of atomically precise clusters which can be activated to give model heterogeneous catalysts. This review has attempted to summarize a large number of activation strategies such that researchers can continue to make fantastic gains in the design and utilization of atom-precise clusters for a wide range of catalytic reactions of commercial interest.

## Conflicts of interest

There are no conflicts of interest to declare.

## Supplementary Material
